# Prevalence, Incidence, Prognosis, Early Stroke Risk, and Stroke-Related Prognostic Factors of Definite or Probable Transient Ischemic Attacks in China, 2013

**DOI:** 10.3389/fneur.2017.00309

**Published:** 2017-06-30

**Authors:** Bin Jiang, Haixin Sun, Xiaojuan Ru, Dongling Sun, Zhenghong Chen, Hongmei Liu, Yichong Li, Mei Zhang, Limin Wang, Linhong Wang, Shengping Wu, Wenzhi Wang

**Affiliations:** ^1^Department of Neuroepidemiology, Beijing Neurosurgical Institute, Beijing Tiantan Hospital, Capital Medical University, Beijing, China; ^2^Beijing Municipal Key Laboratory of Clinical Epidemiology, Beijing, China; ^3^National Office for Cerebrovascular Diseases (CVD) Prevention and Control in China, Beijing, China; ^4^National Center for Chronic and Non-communicable Disease Control and Prevention, Chinese Center for Disease Control and Prevention, Beijing, China

**Keywords:** prevalence, incidence, prognosis, prognostic factors, transient ischemic attacks, stroke

## Abstract

The epidemiological characteristics of transient ischemic attacks (TIAs) in China are unclear. In 2013, we conducted a nationally representative, door-to-door epidemiological survey on TIA in China using a complex, multistage, probability sampling design. Results showed that the weighted prevalence of TIA in China was 103.3 [95% confidence interval (CI): 83.9–127.2] per 100,000 in the population, 92.4 (75.0–113.8) per 100,000 among men, and 114.7 (87.2–151.0) per 100,000 among women. The weighted incidence of TIA was 23.9 (17.8–32.0) per 100,000 in the population, 21.3 (14.3–31.5) per 100,000 among men, and 26.6 (17.0–41.7) per 100,000 among women. No difference in average prognosis was found between TIA and stroke in the population. Weighted risk of stroke among TIA patients was 9.7% (6.5–14.3%), 11.1% (7.5–16.1%), and 12.3% (8.4–17.7%) at 2, 30, and 90 days, respectively. The risk of stroke was higher among male patients with a history of TIA than among female patients with a history of TIA (OR: 2.469; 95% CI: 1.172–5.201; *P* = 0.018), and higher among TIA patients with hypertension than among TIA patients without hypertension (OR: 2.671; 1.547–4.613; *P* < 0.001). It can be concluded that there are an estimated 1.35 million TIA patients nationwide, with 0.31 million new cases of TIA annually in China. TIA patients were not better managed prior to a stroke event. Early risk of stroke among TIA patients is high. Sex and hypertension may be stroke-associated prognostic factors among TIA patients. TIA clinics and surveillance should be integrated into the national health-care system.

## Introduction

A transient ischemic attack (TIA) requires special attention because it is a harbinger of a stroke event. Epidemiological data on TIA events in the general population may be different from clinical data from hospital-based studies and better suited for the prevention and control of TIA and stroke in the general population. Regrettably, TIA prevalence and incidence in the general population may be overestimated because many cases of alleged TIA were included in a few population-based studies that did not rely upon a dependable diagnostic standard and in which patients self-reported TIA symptoms (1, 2). In addition, it may be more meaningful to estimate TIA prognosis in the population on the basis of TIA prevalence and incidence in the population. It is unclear whether a difference between TIA and stroke prognosis exists. Furthermore, early stroke risk and stroke-associated prognostic factors in all cases of TIA survivors in the population are also unclear. Accordingly, we investigated the prevalence, incidence, prognosis, early stroke risk, and stroke-associated prognostic factors of TIA in China by traditional epidemiological methods.

## Materials and Methods

### Sampling Design and Participants

**Figure 1:**
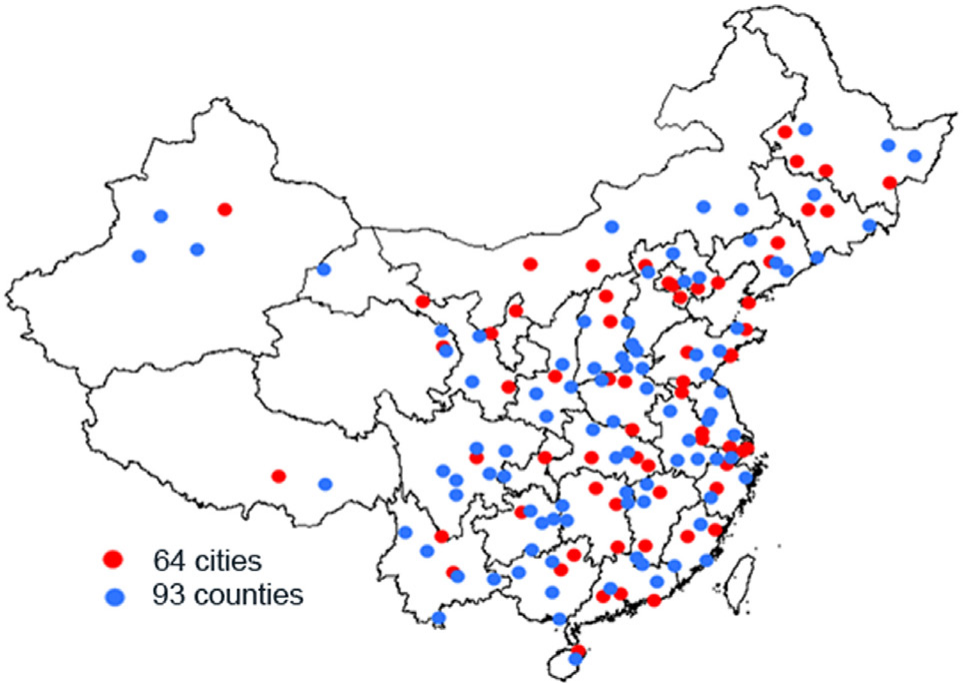
Distribution of survey sites in 31 provinces of China.

A complex, multistage probability sampling design was used to define the sampling frame and the participants (3, 4). The sampling frame for this survey was the National Disease Surveillance Points (DSPs) System with a population of about 74 million people, which may represent the 2010 China national census data with respect to geographical distribution, social status, and economic status. For the determination of the sampling frame, the primary cluster unit (PSU) was cities in urban areas and counties in rural areas. The 2010 Census data and the probability proportionate to population size sampling (PPS) were used to select 64 cities and 93 counties across 31 provinces of China (i.e., 157 DSPs or survey sites in Figure 1).

The sample size for this survey was calculated at approximately 600,000 subjects based on a 1% TIA prevalence, which was postulated on the base of increased risk factors in population and a 6.2/1,000 TIA prevalence from a previous epidemiological survey on TIA prevalence with the same method in six cities in China (5), with two-sided estimates of 95% CI, a design effect (deff) value of 5, and a relative error of 15%, according to the complex sampling formula as follows:

$$N=\text{deff}\frac{u^{2}p(1-p)}{d^{2}}$$

In the first stage of sampling within the sampling frame, the unit of sampling was a “neighborhood” (Jiedao) within cities, or “townships” (Xiang) in rural areas. Both “Jiedao” and “Xiang” represent communities with a primary government and a population ranging from 30,000 to 100,000 people. PPS sampling was again used for the selection of units at this stage, such that the probability of selection was based on the population size of the neighborhood or township. In the second stage of sampling, one or more neighborhood committees (administrative villages) with a total population of at least 4,500 residents (approximately 1,500 households) were selected from the sampled neighborhoods (townships) in each sites using random cluster sampling. The participants were people who had lived in the county (or district) for at least 6 months in the past year. Families and family members who refused to participate were not replaced.

In this retrospective epidemiological survey, TIA point prevalence was defined as the rate of a first-time TIA in the life time of an individual among the survival people prior to midnight on August 31, 2013 from the sampled families. TIA incidence was defined as the rate of the first TIA within a year among the survival population prior to midnight on August 31, 2012 from the sampled families. For this survey, 602,715 people were evaluated in 155 DSPs, with a response rate of 80.8% among 745,588 people.

### Preparations, Case Ascertainment, and Quality Assurance

In the beginning of this survey, an operational manual was widely discussed with a variety of experts including epidemiologists, neurologists from around the world, and senior practitioners from the Centers for Disease Control and Prevention (CDC) in 2012; this manual was further modified according to the findings of pilot investigations performed in Chaoyang District, Beijing and Xingtang County, Hubei Province in 2013. National training sessions for trainers and provincial training sessions for investigators were held in 2013 before the survey. Simultaneously, the sample population to be surveyed was chosen from 157 DSPs across 31 provinces. In the defined sample population, the organization and mobilization of the survey were performed by provincial and local CDCs along with health and administration authorities.

**Figure 2:**
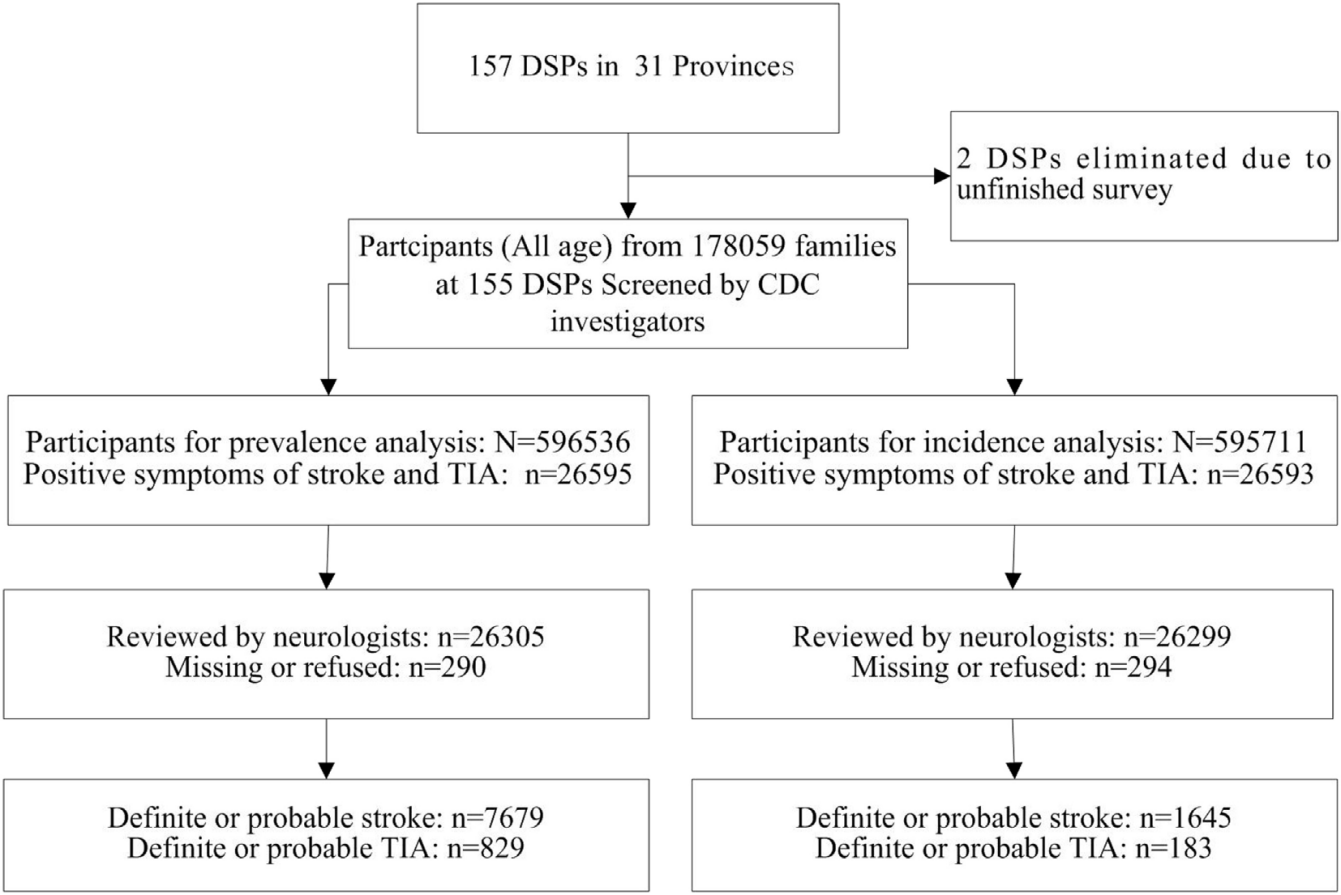
Flowchart for stroke and transient ischemic attack (TIA) case ascertainment. Note: DSPs, disease surveillance points; CDCs, centers for disease control and prevention.

From 1 September 2013 through 31 December 2013, CDC investigators visited each household in the sample population to collect the participants’ signed informed consent forms and complete the preliminary screening form, including basic information about family members, family deaths since 1st January 2012, and positive TIA or stroke symptoms experienced by family members. Participants who had positive TIA or stroke symptoms were invited to see a neurologist in a town/village clinic. Definite and probable TIA or stroke diagnoses were determined after the neurologist completed a physical examination and reviewed previous medical documents, including cardio-cerebro-vascular-related medical records, computed topography (CT)/magnetic resonance imaging (MRI) scans, and an identity card or household register. In this survey, participants with suspected stroke or TIA symptoms were identified by CDC investigators and interviewed by neurologists. Ultimately, 829 cases of first-ever TIAs were diagnosed by neurologists. Among them, 183 cases of first-ever TIAs occurred between September 1, 2012 and August 31, 2013 (see Figure 2).

Quality control was involved in all phases of the survey and supervised preparations, field works, and data processing. Three tiers of a quality control network separately formed by CDC investigators and hospital neurologists, i.e., national, provincial, and local groups of quality control, were established to supervise investigators, organization, and mobilization of the survey, and questionnaire inquiries. All of the questionnaires were mailed to the Beijing Neurosurgical Institute at the end of the investigation, checked by research staff, and then doubly input into a database by specialized staff according to a strict procedure. In this survey, two DSPs were excluded because the investigation had poor quality or was incomplete.

### Diagnostic Criteria

In this epidemiological study, a TIA was defined as the sudden onset of a focal neurologic symptom or sign lasting less than 24 h, presumably brought on by a transient decrease in blood supply that rendered the brain ischemic in the area producing the symptom (6). A definite TIA was defined as the sudden onset of transient limb paralysis, with or without other signs, lasting up to 24 h and leaving no significant deficits. A probable TIA was defined by other transient focal neurologic deficits lasting up to 24 h (7). Possible TIAs with an ambiguous history of symptoms, non-focal symptoms, or vague for anxiety or emotional symptoms were excluded because too many cases were found that could not be properly diagnosed in a population-based study.

The minimum criterion for definite or probable stroke was evidence of a sudden or rapid onset of neurological symptoms lasting for >24 h or leading to death without evidence of a non-stroke cause (8).

### Ethical Approval

This study was approved by the Ethics Committee of the Beijing Tiantan Hospital affiliated with the Capital Medical University, shared by the Beijing Neurosurgical Institute. Informed consent was obtained from all participants or their caregivers. This study was performed in accordance with the Declaration of Helsinki.

### Statistical Analysis

Statistical analyses were conducted on weighted data to account for the complex sampling designs. Weighted coefficients were calculated by considering sampling weights, non-response weights, and post-stratification weights to obtain the national estimates using SAS^®^ software version 9.4 (SAS Institute Inc., Cary, NC, USA). Population information from the 2010 China census data was used to calculate post-stratification weights.

Estimates of prevalence, incidence, and 95% confidence intervals (CIs) of TIAs were computed by age and sex groups. The prognosis for TIA in the population was estimated based on the prevalence and incidence of TIA in the population, which was different from the prognosis estimates in the TIA cohort. Prognostic factors associated with stroke in TIA patients were determined using complex samples logistic regression analyses after adjusting for different explanatory factors. The explanatory risk factors included: age group (25–34/≥35–44/45–54/55–64/65–74/75–84/≥85); sex (men/women); duration of TIA (≤10/10 min to 1/≥1 h/unknown); ethnicity (Han ethnicity/other ethnicity); education (primary school/middle school/college and higher); marriage status (single or windowed/married); occupation (retiree or homemaker/farmer/worker or employee or entrepreneurs); hypertension (defined as having a history of hypertension, or taking antihypertensive medication in the recent 4 weeks, having a systolic blood pressure ≥140 mm Hg or having a diastolic blood pressure ≥90 mm Hg; yes/no); history (yes/no/unknown) of diabetes mellitus, hyperlipidemia, atrial fibrillation, and coronary heart disease; smoking (regular smoking/occasional smoking/quit smoking/never smoked/unknown); drinking (regular drinking/occasional drinking/quit drinking/never drank/unknown). All of these statistical calculations on complex samples were performed using SPSS 15.0 software (SPSS Inc., Chicago, IL, USA). *P* < 0.05 was considered statistically significant.

## Results

The characteristics of the study sample from the national epidemiological survey of TIAs in China, 2013 are shown in Table 1. Among the 596,536 people evaluated for the prevalence analysis, 829 cases (326 definite plus 503 probable) of first-ever TIAs in a lifetime were investigated on August 31, 2013 (see Table 2; Tables S1 and S2 in Supplementary Material). Among the 595,711 people assessed for the incidence analysis, 183 (82 definite and 101 probable) first-ever TIAs were found between September 1, 2012 and August 31, 2013 (see Table 3; Table S2 in Supplementary Material).

**Table 1:** Characteristics of the study sample from the national epidemiological survey of transient ischemic attacks (TIAs) in China, 2013.

Characteristic	Prevalence			Incidence		
	No.	%	Weighted^a^ (%)	No.	%	Weighted^a^ (%)
**Age group**						
0–24	160,382	26.9	33.8	166,840	28.0	34.7
25–34	91,435	15.3	14.9	89,597	15.0	15.1
35–44	99,582	16.7	18.3	102,999	17.3	18.5
45–54	93,763	15.7	13.9	90,667	15.2	13.4
55–64	80,155	13.4	10.5	78,075	13.1	10.2
65–74	44,840	7.5	5.5	43,245	7.3	5.3
75–84	22,200	3.7	2.6	20,585	3.5	2.4
≥85	4,179	0.7	0.5	3,703	0.6	0.4
**Sex**						
Men	300,192	50.3	51.1	299,725	50.3	51.1
Women	296,344	49.7	48.9	295,986	49.7	48.9
**Ethnicity**						
Han	521,343	87.4	91.8	518,151	87.0	91.4
Other	75,193	12.6	8.2	77,560	13.0	8.6
**Education, *n* (%)**						
Primary school	248,916	41.7	39.6	245,192	41.2	38.9
Middle school	294,209	49.3	49.0	294,193	49.4	49.2
College and higher	51,730	8.7	11.2	51,721	8.7	11.3
Missing	1,681	0.3	0.2	4,605	0.8	0.5
**Marital status, *n* (%)**						
Married	391,160	65.6	60.9	391,124	65.7	61.2
Single	116,817	19.6	24.0	115,438	19.4	23.8
Widowed	32,734	5.5	4.2	32,731	5.5	4.2
Other	53,735	9.0	10.6	51,402	8.6	10.2
Missing	2,090	0.4	0.2	5,016	0.8	0.6
**Occupation, *n* (%)**						
Students	108,978	18.3	23.1	105,510	17.7	22.4
Worker	45,021	7.5	8.7	45,004	7.6	8.8
Farmer	271,068	45.4	38.6	270,916	45.5	38.8
Employee	46,676	7.8	9.8	46,674	7.8	9.9
Entrepreneurs	52,518	8.8	10.2	52,516	8.8	10.2
Retiree or homemaker	66,169	11.1	8.7	66,145	11.1	8.7
other	4,439	0.7	0.7	4,356	0.7	0.7
Missing	1,667	0.3	0.2	4,590	0.8	0.5
**Place of residence**						
Urban	248,570	47.4	52.9	248,090	47.4	52.8
Rural	347,966	52.6	47.1	347,621	52.6	47.2
**Geographic location**						
Eastern China	201,354	33.8	40.7	201,196	33.8	40.7
Central China	239,735	40.2	32.0	239,288	40.2	32.0
Western China	155,447	26.1	27.3	155,227	26.1	27.3

*^a^Complex sample weights were used to obtain nationally representative estimates*.

**Table 2:** Prevalence^a^ of transient ischemic attack (TIA) in China, 2013 (1/100,000 person*life time).

Age group	Men				Women				Total	
	Population	TIA	Prevalence^b^	95% CI^b^	Population	TIA	Prevalence^b^	95% CI^b^	Prevalence^b^	95% CI^b^
0–24	83,953	0	0	0	76,429	0	0	0	0.0	0
25–34	45,020	3	3.0	0.8–11.8	46,415	3	2.2	0.3–14.8	2.6	0.8–8.1
35–44	50,759	11	14.4	6.9–30.1	48,823	19	24.5	13.5–44.5	19.3	11.2–33.3
45–54	46,879	55	112.9	74.7–170.4	46,884	82	144.8	93.5–224.1	128.5	93.1–177.4
55–64	39,366	126	310.7	231.2–417.5	40,789	140	409.3	244.7–683.8	359.5	260.2–496.5
65–74	21,902	134	551.1	401.6–755.7	22,938	131	555.4	410.8–750.5	553.2	433.9–705.1
75–84	10,545	61	490.4	333.5–720.4	11,655	54	473.0	308.3–724.9	480.9	350.9–658.9
≥85	1,768	3	147.9	38.3–569.3	2,411	7	177.7	68.2–462.7	166.6	76.1–364.2
Total	300,192	393	92.4	75.0–113.8	296,344	436	114.7	87.2–151.0	103.3	83.9–127.2
Subtotal (≥25)	216,236	393	141.7	115.1–174.3	219,915	436	170.9	128.7–226.9	156.2	126.0–193.6

*^a^A point prevalence in a life time, on 31 August 2013*.

*^b^Prevalences and 95% CIs were estimated with complex sample weights*.

**Table 3:** Incidence^a^ of transient ischemic attack (TIA) in China, 2013 (1/100,000 person*years).

Age group	Men				Women				Total	
	Population	TIA	Incidence^b^	95% CI^b^	Population	TIA	Incidence^b^	95% CI^b^	Incidence^b^	95% CI^b^
0–24	86,757	0	0	0	80,083	0	0	0	0	0
25–34	44,351	0	0	0	45,246	2	2.2	0.3–14.9	1.1	0.2–7.4
35–44	52,561	3	3.9	1.1–13.8	50,438	6	7.9	3.2–19.4	5.9	2.8–12.2
45–54	45,289	17	31.7	16.8–59.7	45,378	20	54.8	26.7–112.2	43.0	24.7–74.8
55–64	38,189	34	95.0	57.4–157.3	39,886	29	82.3	45.8–148.0	88.7	61.8–127.4
65–74	21,283	23	97.3	52.9–179.0	21,962	31	142.0	78.6–256.4	119.6	79.8–179.3
75–84	9,757	9	80.6	34.0–191.0	10,828	9	47.3	23.4–95.6	62.5	34.7–112.4
≥85	1,538	0	0.0	0	2,165	0	0.0	0	0.0	0
Total	299,725	86	21.3	14.3–31.5	295,986	97	26.6	17.0–41.7	23.9	17.8–32.0
Subtotal (≥25)	212,968	86	33.0	22.3–48.8	215,903	97	40.3	25.5–63.6	36.6	27.1–49.4

*^a^An incidence in a year from 1 September 2012 to 31 August 2013*.

*^b^Incidences and 95% CIs were estimated with complex sample weights*.

### Prevalence of TIA

**Figure 3:**
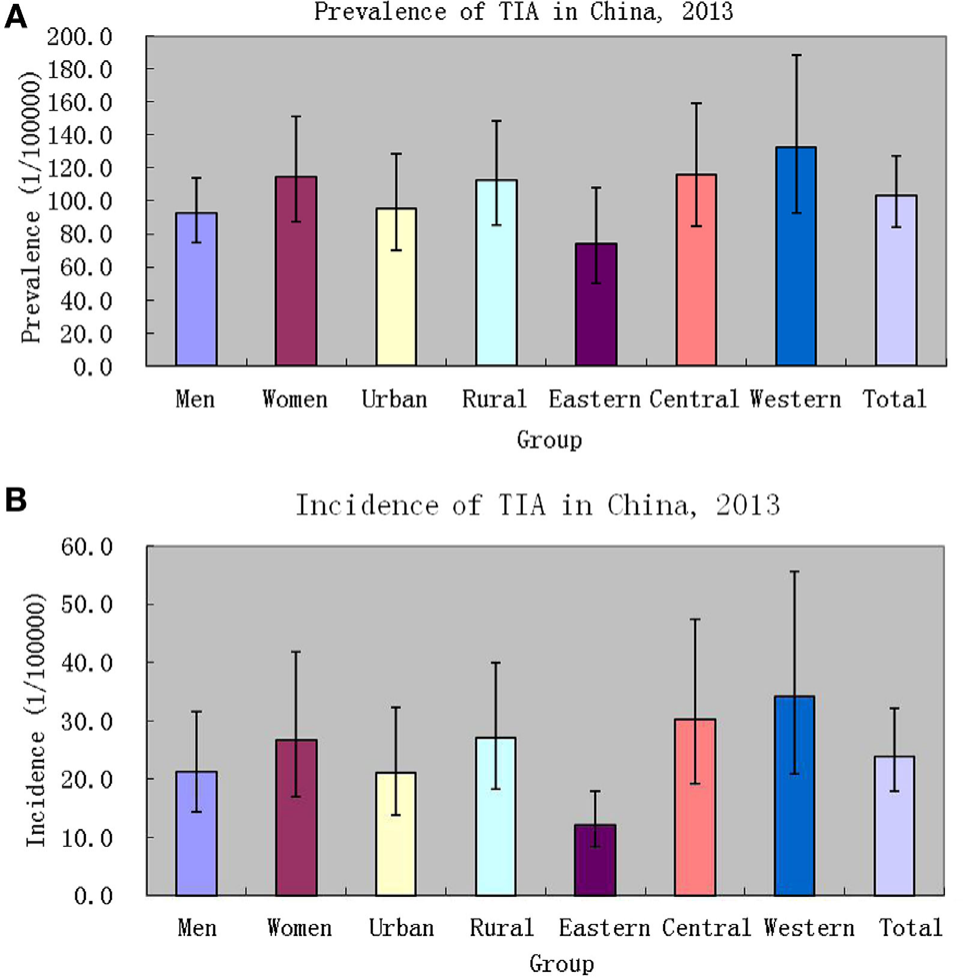
Prevalence [**(A)**, 1/100,000 person*life time] and incidence [**(B)**, 1/100,000 person*years] of transient ischemic attack (TIA) in China, 2013.

In China, the weighted prevalence of TIA was 103.3 (95% CI: 83.9–127.2) per 100,000 in the population, 92.4 (95% CI: 75.0–113.8) per 100,000 among men, and 114.7 (95% CI: 87.2–151.0) per 100,000 among women; 95.0 (95% CI: 70.1–128.8) per 100,000 among urban residents, and 112.6 (95% CI: 85.4–148.5) per 100,000 among rural residents; 73.7 (95% CI: 50.2–108.3) per 100,000 among eastern Chinese, 116.2 (95% CI: 84.7–159.5) per 100,000 among central Chinese, and 132.4 (95% CI: 92.9–188.5) per 100,000 among western Chinese (see Table 2, Figure 3). According to the above-estimated prevalence, there were an estimated 1,353,738 (95% CI: 979,093–1,728,384) TIA patients in the population with 619,184 (95% CI: 462,437–775,931) male TIA patients and 734,555 (95% CI: 482,160–986,949) female TIA patients in China (see Table 2).

### Incidence of TIA

In China, the weighted incidence of TIA was 23.9 (95% CI: 17.8–32.0) per 100,000 in the population, 21.3 (95% CI: 14.3–31.5) per 100,000 among men, and 26.6 (95% CI: 17.0–41.7) per 100,000 among women; 21.1 (95% CI: 13.8–32.3) per 100,000 among urban residents, and 27.0 (95% CI: 18.2–39.9) per 100,000 among rural residents; 12.2 (95% CI: 8.3–17.9) per 100,000 among eastern Chinese, 30.2 (95% CI: 19.2–47.7) per 100,000 among central Chinese, and 34.0 (95% CI: 20.8–55.7) per 100,000 among western Chinese (see Table 3; Figure 3). Marginally statistical difference in the incidence of TIA was found between eastern and central or western Chinese. According to the above-estimated incidence, there were an estimated 311,295 (95% CI: 200,790–421,800) TIA patients annually in the population, with 141,666 (95% CI: 83,218–200,113) male TIA patients and 169,630 (95% CI: 83,393–255,866) female TIA patients in China (see Table 3).

### Prognosis for TIA in the Population

In China, the average prognosis for TIA in the population was estimated to be 4.326 (95% CI: 3.524–5.310) years based on estimates of point prevalence in a lifetime and the annual incidence of TIA, whereas the average prognosis for a stroke in the population was approximately 4.567 (95% CI: 4.173–4.999) years. No prognostic difference between TIA and stroke in the population was found.

### Early Risk of Stroke

Weighted risk of stroke among TIA patients was 9.7% (6.5–14.3%), 11.1% (7.5–16.1%), and 12.3% (8.4–17.7%) at 2, 30, and 90 days, respectively; whereas that among TIA patients with stroke was 52.0% (38.8–65.0%), 59.2% (44.6–72.3%), and 65.6% (50.5–78.1%) at 2, 30, and 90 days, respectively. Given that TIA cases occurred between June 1, 2013 and August 31, 2013 might be right-censored, sensitivity analyses after excluding these cases showed that weighted risk of stroke among TIA patients was 9.9% (6.5–14.6%), 11.0% (7.4–16.0%), and 12.2% (8.3–17.6%) at 2, 30, and 90 days, respectively; whereas that among TIA patients with stroke was 51.7% (37.5–65.7%), 57.6% (42.6–71.4%), and 64.1% (48.5–77.3%) at 2, 30, and 90 days, respectively.

### Stroke-Associated Prognostic Factors

Table 4 shows stroke-associated prognostic factors of TIAs. The risk of stroke was higher among male patients with a positive TIA history than among female patients with a positive history of TIA (OR: 2.469; 95% CI: 1.172–5.201; *P* = 0.018). The risk was also higher among TIA patients with hypertension than among TIA patients without hypertension (OR: 2.671; 95% CI: 1.547–4.613; *P* < 0.001).

**Table 4:** Stroke-associated prognostic factors of transient ischemic attack (TIA) in China, 2013.

Factors	TIA no.	TIA no. with stroke	%	Weighted^a^ (%)	OR	95% CI	*P*-value
**Age group**							
25–34	6	1	16.7	0.9	0.047	0.001–1.700	0.094
35–44	30	3	10.0	14.2	0.693	0.056–8.526	0.773
45–54	137	28	20.4	21.5	0.945	0.132–6.761	0.955
55–64	266	77	28.9	22.5	0.964	0.144–6.463	0.970
65–74	265	92	34.7	29.5	1.164	0.170–7.962	0.877
75–84	115	38	33.0	26.7	0.783	0.101–6.089	0.814
≥85	10	2	20.0	20.3	Reference	Reference	
**Sex**							
Men	393	134	34.1	31.8	2.469	1.172–5.201	0.018
Women	436	107	24.5	18.3	Reference	Reference	
**Duration of TIA**							
≥1 h	128	33	25.8	22.3	0.959	0.491–1.875	0.903
10 min to 1 h	259	86	33.2	26.9	1.388	0.742–2.598	0.304
≤10 min	402	107	26.6	22.5	Reference	Reference	
**Ethnicity**							
Han ethnicity	705	223	31.6	26.7	3.053	0.876–10.636	0.079
Other	124	18	14.5	8.6	Reference	Reference	
**Education**							
Middle school	314	109	34.7	30.8	1.291	0.741–2.250	0.366
College and higher	28	13	46.4	21.0	1.098	0.388–3.107	0.859
Primary school	487	119	24.4	20.7	Reference	Reference	
**Marriage status**							
Married	679	204	30.0	23.2	0.459	0.207–1.018	0.055
Single/windowed	150	37	24.7	31.5	Reference	Reference	
**Occupation**							
Worker/employee/entrepreneurs	75	18	24.0	24.3	1.115	0.488–2.548	0.795
Farmer	469	106	22.6	20.2	0.723	0.417–1.252	0.246
Retiree or homemaker	285	117	41.1	33.0	Reference	Reference	
**Disease history**							
**Hypertension**
Yes	562	195	34.7	29.4	2.671	1.547–4.613	0.000
No	267	46	17.2	12.0	Reference	Reference	
**Diabetes mellitus**
Yes	121	46	38.0	30.9	1.024	0.508–2.066	0.947
No	613	170	27.7	22.9	Reference	Reference	
**Dyslipidemia**
Yes	204	80	39.2	37.9	1.073	0.599–1.923	0.812
No	398	118	29.6	22.8	Reference	Reference	
**Atrial fibrillation**
Yes	35	7	20.0	17.4	0.454	0.142–1.455	0.183
No	647	201	31.1	24.7	Reference	Reference	
**Coronary heart disease**
Yes	148	60	40.5	35.2	1.461	0.799–2.672	0.217
No	531	146	27.5	22.7	Reference	Reference	
**Smoking**
Regular smoking	149	47	31.5	30.0	0.899	0.358–2.254	0.819
Occasional smoking	97	20	20.6	15.2	0.692	0.257–1.864	0.465
Quit smoking	102	45	44.1	40.7	0.861	0.336–2.203	0.754
Never smoked	481	129	26.8	22.2	Reference	Reference	
**Drinking**
Regular drinking	71	17	23.9	30.2	0.986	0.360–2.699	0.978
Occasional drinking	172	33	19.2	14.1	0.487	0.192–1.235	0.129
Quit drinking	99	44	44.4	46.7	1.843	0.809–4.201	0.145
Never drank	487	147	30.2	23.7	Reference	Reference	

*^a^Complex sample weights were used to obtain nationally representative estimates*.

## Discussion

Transient ischemic attack case ascertainment and diagnosis are major challenges in epidemiological surveys of TIA. Regarding the diagnostic criteria for TIA in the population, we still defined TIA as the sudden onset of a focal neurologic symptom or sign lasting less than 24 h, presumably brought on by a transient decrease in blood supply causing brain ischemia in the area producing the symptom. This definition is endorsed in the epidemiological survey by 2009 guidelines from the American Heart Association and American Stroke Association (AHA/ASA) for better practice and comparison with other previous epidemiological surveys (6). In contrast to other previous studies, we concurrently investigated TIA and stroke cases in the population with the discriminating factor of a time window of 24 h for focal neurologic symptom or sign duration rather than using a CT or MRI check-up because the former can apply to all cases of TIA or stroke in the population. Furthermore, a higher rate (e.g., 19.9% in this survey) of silent lacunar cerebral infarction in the population with a CT or MRI check-up also contrasts with using CT or MRI check-ups in the epidemiological survey. Additionally, cases of self-reported TIA symptoms from other studies were excluded from the possible TIA category because it was too difficult to diagnose these cases due to the lack of exclusive symptoms and signs in TIA, particularly in the posterior circulation. Only definite or probable TIA diagnoses were included in this survey based on TIA event progression, as defined by neurologists, from all cases with positive stroke or TIA symptoms in the preliminary screening form. A previous study found that nearly half of the subjects who experienced a transient neurological attack (TNA) had symptoms that were not entirely typical for a TIA. The differences in the associations of risk factors with typical TIA and non-specific TNA suggest an alternative underlying mechanism for the symptoms that may lead to different ancillary investigations and potential treatment (9).

**Figure 4:**
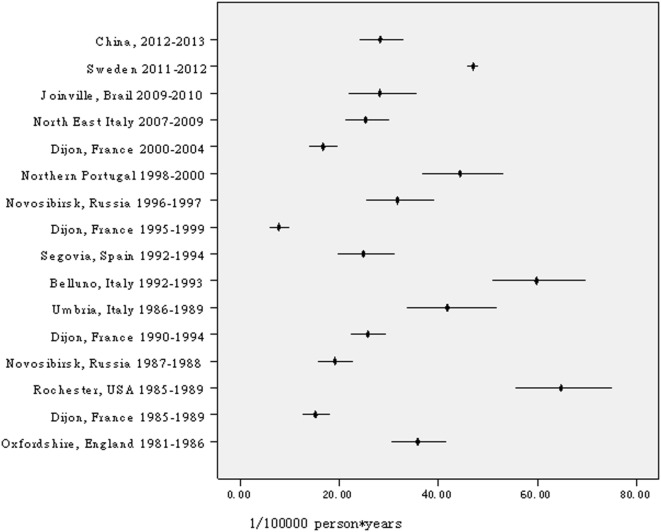
Annual incidence of transient ischemic attack per 100,000 people per year in different all-age populations, age standardized to the European population.

Currently, this is the largest-scale sampling survey on TIA in the world, including approximately 600,000 people. In China, a survey on the prevalence and incidence of TIA included 63,195 participants in six cities but was conducted 30 years ago (5). Another survey on prevalence included 98,658 participants nationwide and conducted 3 years ago (2). After age was standardized to the same corresponding population, the prevalence in this survey was lower than that found in previous surveys (1, 2, 5, 7). With regard to prevalence, a study from Japan found that the prevalence ratio of TIA in Japan is lower by approximately one-third to one-half of that in Western countries (10). Similar findings for prevalence were found in our studies and another previous study in China (5). However, two studies, one from Korea (11) and another from China (2), challenged the above impression. According to these studies, the prevalence of TIA in Asian people was not lower than that in Western countries (2, 11, 12). According to our survey, the incidence of TIA in China is higher than that in Dijon (France) during the periods of 1985–1989, 1995–1999, and 2000–2004 (13), in the IBERICTUS Study (Spain) (14), or in Novosibirsk (Russia) during the period of 1987–1988 (15), with results close to that in OCSP (England) (16), in Dijon (France) during the period of 1990–1994 (13, 17), in Segovia (Spain) (18), in Novosibirsk (Russia) during the period of 1996–1997 (15), in North East Italy (19), and in Joinville (Brazil) (20), but lower than those in Rochester (NY, USA) (21) or the Greater Cincinnati/Northern Kentucky (USA) (22), Umbria (Italy) (23) or Belluno (Italy) (24), Porto and UP Aguiar (Northern Portugal) (25), Aarhus (Denmark) (26), Sweden (27), and Auckland (New Zealand) (28). Figure 4 shows the annual incidence of TIA per 100,000 people per year in different all-age populations, with age standardized to the European population using Poisson distribution. Differences in rates of prevalence and incidence of TIA may be attributed to diverse experimental designs (including time-point stipulation for prevalence, exclusion of recurrent TIA for incidence, and prospective surveillance or retrospective surveys etc.), case ascertainment, diagnosis, and time period of the survey. Exploring demographic differences in TIA prevalence and incidence across populations calls for further studies using standard designs, case ascertainment, diagnosis, and concurrent implementation. However, difference in the incidence of TIA between eastern and central or western Chinese in this study may be due to difference in control to stroke-related risk factors. As seems to be confirmed by 32.8, 29.8, and 17.9% of hypertension control, respectively, in eastern, central, and western TIA patients with hypertension, although no corresponding data in population.

To our knowledge, this is the first study to infer TIA average prognosis in a population by point prevalence in a lifetime and the annual incidence of TIA. We found that the average prognosis for TIA in the population was 4.326 year (95% CI: 3.524–5.310), which was consistent with the average prognosis for stroke in the population. In other words, a TIA is similar to a stroke from perspective of prognosis in the population; however, TIAs are not appropriately treated before evolving into a stroke. Only 3.0% of 7,679 first-ever stroke patients in this survey had histories of TIA. The rate was lower than that in a previous report (29). This could be partially explained by the definitions of TIA and stroke, because no cases of TIA within 24 h prior to stroke were acknowledged in our study. By contrast, one-third of the 829 first-ever TIA patients developed strokes. This is consistent with the current belief that a third of TIA patients will have a stroke. And moreover, weighted early risk of stroke (6.5–14.3%, 7.5–16.1%, and 8.4–17.7% at 2, 30, and 90 days, respectively) among TIA patients in this study was consistent with pooled risk of stroke (4.9–14.9%, 9.8–17.1%, and 9.3–25.3% at 2, 30, and 90 days, respectively) in three studies with active outcome ascertainment (30). The immediate risk of stroke after a TIA may be underestimated owing to methodological problems in previous studies of prognosis, because whatever hospital-based and population-based cohort studies have reported risks of up to 10% at 7 days and 15% at 30 days (31, 32), similar to our findings. However, 52.0% of strokes occurred within 48 h of the TIA and 53.7% during the 7 days among TIA patients with stroke in this survey, implying that the “warning” time-window of stroke prevention is short and urgent. In the multivariate analyses, only male sex and hypertension were associated with a higher risk of stroke among TIA patients. However, we could not determine whether age, TIA duration, or diabetes in the ABCD^2^ score system (6) was independently associated with stroke in these cases.

The strengths of this study are its professional design, implementation, case ascertainment, and the diagnosis of TIA in this sampling survey. In contrast to other studies (2), replacement response was not allowed. Furthermore, TIA or stroke symptoms screened by a preset, structured questionnaire plus the professional diagnosis of a TIA guarantee the validity of the survey. Neurologists interviewed participants with positive TIA/stroke symptoms and ultimately confirmed 829 cases of TIA with information on the time of onset, duration, disappearance of symptoms, and a detailed description of symptoms confirming attributes of prevalence and incidence according to standards. However, a recall bias existed in this cross-sectional survey because we could not obtain accurate TIA information on dead cases within the defined periods of incidence, even though we examined all deaths in the period. Indeed, it was also difficult for elderly subjects to accurately recall an episode of TIA before having a stroke in their lifetimes. Contrary to evidence of increased prevalence and incidence of stroke with age, the prevalence and incidence of TIA decreased only in subjects of both sexes aged 75 years or older.

In conclusion, it is estimated that there are 1.35 millions of TIA patients in total and 0.31 million new cases of TIA annually in China. TIA patients did not routinely receive appropriate treatment before the development of a stroke. Early risk of stroke among TIA patients is high. Sex and hypertension may be stroke-associated prognostic factors among TIA patients. TIA clinics and surveillance should be integrated into the national health care system.

## The Program Working Group for the National Epidemiological Survey of TIAs in China

The following investigators participated in this study: *Steering Committee*: B. Jiang (principal investigator), WZ. Wang (co-principal investigator), H. Sun, X. Ru, D. Sun, Z. Chen, H. Liu, Y. Li, M. Zhang, Limin Wang, Linhong Wang, S. Wu.

## Participating CDCs and Principal Staff in the Screening Group

**Gang Zhou**, Henan Provincial Center for Disease Control and Prevention; **Guide Song**, Tianjin Centers for Disease Control and Prevention; **Guowei Pan**, Liaoning Provincial Center for Disease Control and Prevention; **GuoxiaBai**, Tibet Autonomous Region Center for Disease Control and Prevention; **Hong Yang**, Guangxi Zhuang Autonomous Region Center for Disease Prevention and Control; **Hongmei Wang**, Hainan Center for Disease Control and Prevention; **Huilin Liu**, Hunan Provincial Center for Disease Control and Prevention; **Jingang Ma**, Shaanxi Center for Disease Control and Prevention; **Junqing Zhu**, Hebei Provincial Center for Disease Control and Prevention; **Laixin Liu**, The Center for Disease Control and Prevention of Xinjiang Uygur Autonomous Region; **Liping Zhu**, Jiangxi Province Center for Disease Control and Prevention; **Min Yu**, Zhejiang Provincial Center for Disease Control and Prevention; **Ming Wu**, Jiangsu Provincial Center for Disease Control and Prevention; **Minru Zhou**, Qinghai Center for Disease Prevention and Control; **Pengfei Ge**, Gansu Center for Disease Control and Prevention; **Qingjun Zhang**, Hubei Center for Disease Prevention and Control; **Qingping Shi**, Yunnan Centers for Disease Control and Prevention; **Qingsheng Wu**, Anhui Center for Disease Control and Prevention; **Tao Liu**, Guizhou Provincial Center for Disease Control and Prevention; **Xianbin Ding**, Chongqing Center for Disease Prevention and Control; **Xiaolei Guo**, Shandong Center for Disease Control and Prevention; **Xinjian Li**, Shanghai Municipal Center for Disease Control and Prevention; **Xue Zhou**, Heilongjiang Provincial Center for Disease Control and Prevention; **Yanjun Xu**, Guangdong Provincial Center for Disease Control and Prevention; **Yine Zhang**, NingXia Center For Diseases Control and Prevention; **Ying Deng**, Sichuan Center for Disease Control and Prevention; **Ying Li**, Center for Disease Control and Prevention, No. 2 Agriculture Division of Xinjiang Production and Construction Corps.; **Ying Ye**, Fujian Center for Disease Control and Prevention; **Yingli Zhu**, The Center for Disease Control and Prevention of Jilin Province; **Yonggang Qian**, Inner Mongolia Autonomous Region Center for Disease Control and Prevention; **Zeping Ren**, Center for Disease Control and Prevention of Shanxi Province; **Zhong Dong**, Beijing Center for Disease Control and Prevention.

## Participating Hospitals and Principal Staff in the Diagnosing Group

**Chao Qin**, The First Affiliated Hospital of Guangxi Medical University; **Dengji Pan**, Tongji Hospital, Tongji Medical College, Huazhong University of Science and Technology; **Guohua Zhang**, Hospital of Inner Mongolia Medical College; **Jiachun Feng**, The First Hospital, Jilin University; **Jinghua Wang**, Tianjin Medical University General Hospital; **Jinsheng Zeng**, The First Affiliated Hospital, Sun Yat-Sen University; **Kai Wang**, The First Affiliated Hospital of Anhui Medical University; **Li Guo**, The Second Hospital of Hebei Medical University; **Li He**, West China Hospital, Sichuan University; **Liming Zhang**, The First Hospital of Harbin Medical University; **Meiping Ding**, The Second Affiliated Hospital of Zhejiang University School of Medicine; **Ning Wang**, The First Affiliated Hospital of Fujian Medical University; **Oumei Cheng**, The First Affiliated Hospital, Chongqing Medical University; **Shengnian Zhou**, Qilu Hospital of Shandong University; **Shizheng Wu**, Qinghai Provincial People’ s Hospital; **Shurong Wang**, Hainan Medical University Affiliated Hospital; **Xiaomu Wu**, Jiangxi Provincial People’s Hospital; **Xiaoning Zhang**, The First Teaching Hospital of Xinjiang Medical University.; **Xiaoyi Li**, Guizhou Provincial Pepole’s Hospital; **Xiaoyuan Niu**, First Hospital, Shanxi Medical University; **Xingquan Zhao**, Beijing Tiantan Hospital, Capital Medical University; **Xuedong Liu**, Xijing Hospital, The Fourth Military Medical University; **Yanhui Du**, The General Hospital of Ningxia Medical University; **Yingdong Zhang**, Nanjing First Hospital, Nanjing Medical University; **Yuhong Zhu**, The Second Affiliated Hospital of Kunming Medical University; **Yuming Xu**, The First Affiliated Hospital of Zhengzhou University; **Yunhai Liu**, Xiangya Hospital, Central South University; **Zhen Hong, Ding Ding**, Huashan Hospital, Fudan University; **Zhenghong Shi**, Lanzhou University Second Hospital; **Zhi Pu**, The People’s Hospital of Tibet Autonomous Region; **Zhiyi He**, First Affiliated Hospital of China Medical University.

## Author Contributions

BJ and WW were the principal investigators responsible for the survey. All authors contributed to the study design, its implementation and field works, data collection, and analysis. BJ performed the statistical analysis and manuscript writing. All contributors discussed the findings and approved the final version for publication.

## Conflict of Interest Statement

The authors declare that the research was conducted in the absence of any commercial or financial relationships that could be construed as a potential conflict of interest.
