# Supplementary material for: Prevalence, Incidence, Prognosis, Early Stroke Risk, and Stroke-Related Prognostic Factors of Definite or Probable Transient Ischemic Attacks in China, 2013
Source: Front Neurol. 2017 Jun 30;8:309. doi: 10.3389/fneur.2017.00309 (PMC5491639; doi:10.3389/fneur.2017.00309)
Supplement: Supplementary file 1 [file Table_1.PDF]

Supplemental Table 1 Prevalence and treatment status of major chronic diseases or risk factors in the 829 TIA patients

	Prevalence	Weighted[a] prevalence	Rate of treatment	Weighted[a] rate of treatment
Hypertension	67.8% (562/829)	71.7%(970381/1353738)	68.9%(387/562)	63.0%(611011/970381)
Diabetes	14.6%(121/829)	12.8%(171580/1353738)	78.5%(95/121)	81.4%(139675/171580)
Dyslipidaemia	24.6%(204/829)	22.8%(308297/1353738)	51.5%(105/204)	42.9%(132391/308297)
Atrial firbillation (AF)	4.2%(35/829)	3.7%(50733/1353738)	54.3%(19/35)	65.8%(33388/50733)
Cororary heart diseases (CHD)	17.9%(148/829)	13.7%(186348/1353738)	-	-
CHD+Hypertension	76.4%(113/148)	85.7%(159642/186438)	76.1%(86/113)	73.0%(116487/159642)
CHD+Diabetes	23.6%(35/148)	26.2%(48749/186348)	71.4%(25/35)	68.6%(33455/48749)
CHD+Dyslipidaemia	35.8%(53/148)	41.0%(76432/186348)	50.9%(27/53)	51.6%(39402/76432)
CHD+AF	10.1%(15/148)	9.8%(18345/186348)	53.3%(8/15)	48.5%(8899/18345)
Current smoking	29.7%(246/829)	32.0%(432880/1353738)	-	-
Quit smoking	-	-	29.3%(102/348)	22.6%(126562/559442)
Alcohol drinking	29.3%(243/829)	29.7%(402514/1353738)	-	-
Quit drinking	-	-	28.9%(99/342)	25.8%(139821/542335)

a Complex sample weights were used to obtain nationally representative estimates.
