# Supplementary material for: Prevalence, Incidence, Prognosis, Early Stroke Risk, and Stroke-Related Prognostic Factors of Definite or Probable Transient Ischemic Attacks in China, 2013
Source: Front Neurol. 2017 Jun 30;8:309. doi: 10.3389/fneur.2017.00309 (PMC5491639; doi:10.3389/fneur.2017.00309)
Supplement: Supplementary file 2 [file Table_2.PDF]

Supplemental Table 2 Special examinations in TIA patients for prevalence and incidence analyses

	TIA patients for the prevalence analysis				TIA patients for the incidence analysis			
	No. (829)	%	Weighted[a] No. (1353738)	Weighted[a] %	No. (183)	%	Weighted[a] No. (311295)	Weighted[a] %
CT/CTA	517	62.4%	777462	57.4%	132	72.1%	214455	68.9%
MRI/MRA	195	23.5%	260330	19.2%	51	27.9%	82103	26.4%
DSA	24	2.9%	32653	2.4%	3	1.6%	8480	2.7%
TCD	90	10.9%	147100	10.9%	23	12.6%	51534	16.6%
Duplex color Doppler ultrasound for evluation of carotid / subclavian artery	103	12.4%	175020	12.9%	30	16.4%	64805	20.8%
EEG	49	5.9%	102967	7.6%	9	4.9%	23767	7.6%

a Complex sample weights were used to obtain nationally representative estimates.
