# Supplementary material for: Prevalence, Incidence, Prognosis, Early Stroke Risk, and Stroke-Related Prognostic Factors of Definite or Probable Transient Ischemic Attacks in China, 2013
Source: Front Neurol. 2017 Jun 30;8:309. doi: 10.3389/fneur.2017.00309 (PMC5491639; doi:10.3389/fneur.2017.00309)
Supplement: Supplementary file 3 [file Table_3.PDF]

Supplemental Table 3 CDC investigators and neurologists in 32 provinces and autonomous regions of China

Provinces/regions	CDC investigators	Neurologists
Beijing	18	10
Tianjin	18	10
Hebei Province	83	45
Shanxi Province	63	8
Inner Mongolia Autonomous Region	25	15
Liaoning Province	27	12
Jilin Province	23	14
Heilongjiang Province	80	10
Shanghai	31	6
Jiangsu Province	64	9
Zhejiang Province	80	18
Henan Province	45	23
Anhui Province	32	20
Fujian Province	60	19
Jiangxi Province	58	8
Shandong Province	115	20
Hubei Province	91	6
Hunan Province	37	15
Guangdong Province	82	9
Guangxi Zhuang Autonomous Region	33	18
Guizhou Province	59	13
Hainan Province	20	6
Chongqing	13	4
Sichuan Province	86	8
Yunnan Province	73	10
Tibet Autonomous Region	34	9
Shaanxi Province	83	7
Gansu Province	61	10
Qinghai Province	49	5
Ningxia Hui Autonomous Region	28	8
Xinjiang Uygur Autonomous Region	44	6
Xinjiang Production and Construction Corps	11	2
Total	1626	383

CDC, Centers for Disease Control and Prevention
